# Effect of Lower and Upper Body High Intensity Training on Genes Associated with Cellular Stress Response

**DOI:** 10.1155/2017/2768546

**Published:** 2017-05-15

**Authors:** Małgorzata Żychowska, Andrzej Kochanowicz, Kazimierz Kochanowicz, Jan Mieszkowski, Bartłomiej Niespodziński, Stanisław Sawczyn

**Affiliations:** ^1^Department of Life Sciences, Gdansk University of Physical Education and Sport, Kazimierza Górskiego 1, 80-336 Gdańsk, Poland; ^2^Department of Gymnastics and Dance, Gdansk University of Physical Education and Sport, Kazimierza Górskiego 1, 80-336 Gdańsk, Poland; ^3^Department of Theory of Sport and Human Motorics, Gdansk University of Physical Education and Sport, Kazimierza Górskiego 1, 80-336 Gdańsk, Poland; ^4^Institute of Physical Education, Kazimierz Wielki University, Sportowa 2, 85-091 Bydgoszcz, Poland; ^5^Department of Sport for All, Gdansk University of Physical Education and Sport, Kazimierza Górskiego 1, 80-336 Gdańsk, Poland

## Abstract

This study aimed to compare the effect of upper and lower body high intensity exercise (HIE) on select gene expression in athletes. Fourteen elite male artistic gymnasts (age 20.9 ± 2.6 years; weight 68.6 ± 7.2 kg; fat free mass 63.6 ± 6.7 kg; height 1.70 ± 0.04 m) performed lower and upper body 30 s Wingate Tests (WAnTs) before and after eight weeks of specific HIIT. Two milliliters of blood was collected before and after (5, 30 min, resp.) lower and upper body WAnTs, and select gene expression was determined by PCR. Eight weeks of HIIT caused a significant increase in maximal power (722 to 751 Wat), relative peak power in the lower body WAnTs (10.1 to 11 W/kg), mean power (444 to 464 W), and relative mean power (6.5 to 6.8 W/kg). No significant differences in lower versus upper body gene expression were detected after HIIT, and a significant decrease in the* IL6/IL10* ratio was observed after lower (−2^∧^0.57 *p* = 0.0019) and upper (−2^∧^0.5 *p* = 0.03) WAnTs following eight weeks of HIIT. It is hypothesized that a similar adaptive response to exercise may be obtained by lower and upper body exercise.

## 1. Introduction

High intensity interval training (HIIT) has become increasingly popular in recent years, in both sport and recreation, as it produces results faster in various athletic categories: muscle strength, muscle oxidative capacity, and muscle glycogen content. These results are similar to those obtained by conventional endurance training [1, 2]. Gaesser and Angadi [3] suggested this type of training is better than long duration, moderate-intensity exercise training for improving fitness and inducing beneficial metabolic adaptations. Kaspar et al. [4] reported that the health-promoting effects are similar to those observed by endurance training.

During exercise, changes in genes associated with cellular stress response are relatively common. Genes encoding heat shock proteins (HSP) or interleukins are easily induced by physical exercise due to alterations in oxidative stress, temperature, heat, and metabolic stress [5, 6]. The adaptive effect to exercises decreases proinflammatory and increases anti-inflammatory cytokines [7, 8] and decreases* HSPA1A *mRNA [9]. Previous data regarding high intensity exercise suggests that this type of muscle effort causes metabolic changes on multiple levels, altering the production of interleukins and heat shock proteins [10–12]. It has been postulated that production of pro- and anti-inflammatory proteins accompanies the stress response, regardless of the kind of stressors (e.g., temperature, physical effort) or signalling pathway activation, including the* HSF-1* and* NF*-*kB* pathways [11, 12]. Changes in genes associated with inflammation and HSP show the result of alterations in signalling via these pathways. There is considerable evidence demonstrating the influence of various exercise types on inflammation [13, 14] and gene expression of heat shock proteins [15, 16], thereby mediating the health benefits of episodic and prolonged exercise.

The health-promoting effects of exercise are associated with interleukin production, eliciting the anti-inflammatory response by decreasing inflammation [10] and increasing stress tolerance. Therefore, determining the balance of pro- and anti-inflammatory cytokines after exercise is important, not only in sports, but also in terms of health [13]. Additionally, the type of training is one of many factors that can influence the adaptive effect to exercise. In athletes, changes in gene expression are not as dynamic when compared to sedentary people. Thompson et al. [17] reported a short-term decrease in IL-6 as an adaptive effect to HIIT in sedentary people, while Zwetsloot et al. [1] described a modest systemic inflammatory response to HIIT in active young men. Unfortunately, these data investigating the adaptive effect were considering only lower body exercises.

Changes in gene expression before and after HIIT or high intensity exercise (HIE) have been studied for physical possibilities, especially anaerobic capacity [18, 19]. Single HIE can increase IL-6 protein expression when compared to continuous moderate-intensity exercise [20], while prolonged HIE can improve chronic, systemic inflammation markers and, consequently, decrease IL-6 expression as an adaptive effect to exercise [17]. However, there is not much data examining adaptive changes between lower and upper body exercises caused by HIIT at the gene activation level.

Upper body exercise is very important in many sports, such as gymnastics or judo, and it is also critical to people unable to perform lower body exercises. In this study, gymnasts were selected as model athletes and subjected to HIIT. It is unclear if adaptive changes to exercise are similar to those obtained by the lower body. Only one study is associated with differences in inflammation between the lower and upper body. The authors analyzed IL-6 and IL-10 protein levels after intense judo exercises involving the lower and upper body separately [21]. Their results suggested that, despite the higher performance in the lower body, the inflammatory response did not differ between exercises performed using the upper body. Based on this data, it is possible that changes in physiological and biochemical indicators which influence gene expression are similar to those obtained for the lower body. The response to this type of exercise may be systemic. Thus, the aim of this study was threefold. First, we aimed to determine changes in genes associated with the cellular stress response after HIE performed by the lower and upper body. Next, we wanted to evaluate adaptive changes to HIE after eight weeks of HIIT, and, finally, we aimed to compare adaptive changes as a result of lower and upper body exercises.

## 2. Material and Methods

### 2.1. Ethics Statement

This study was approved by the Bioethics Committee for Clinical Research at the Regional Medical Chamber in Gdańsk and conducted according to the Declaration of Helsinki. All participants gave their written consent to participate in the study and were informed about the purpose and test procedures. Additionally, they were made aware of possibility of withdrawal of consent at any time for any reason.

### 2.2. Participants

Fourteen elite male gymnasts (age 20.9 ± 2.6 years; weight 68.6 ± 7.2 kg; fat free mass 63.6 ± 6.7 kg; height 1.70 ± 0.04 m) volunteered for the study. Participants were instructed to maintain their normal diet during the days leading up to and on the days of testing, and they were asked to refrain from vigorous exercise and avoid caffeine and alcohol consumption during the 48 hours preceding the testing date. Food was not consumed during testing and water was available ad libitum. Gymnasts had 14.2 ± 2.1 years of sports experience. During the research protocol, the gymnasts trained for one or two sessions per day, six days per week (about 24 hours). Each training session included mainly high intensity anaerobic performance (skip; jump; step up; squat jump; sprint with the load; push-ups; pull-up exercises including abdominal muscles, core, and arm strength training; back; and other specialized exercises using various training simulators) and technical skills (floor exercises, pommel horse, rings, vault, parallel bars, and horizontal bar). One specific week of the training period is presented in Table 1.

### 2.3. Experimental Design

**Figure 1:**
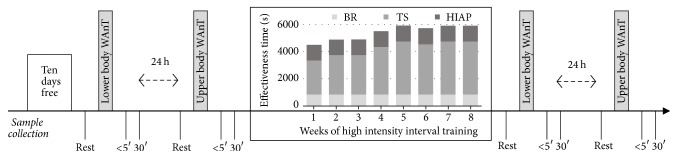
Schematic illustration of the study design. Wingate Anaerobic Test (WAnT), high intensity anaerobic performance (HIAP), technical skills (TS), and biological renewal (BR).

To evaluate anaerobic possibilities and gene expression, the same protocol was used before and after eight weeks of HIIT (Figure 1). Anaerobic components of fitness were determined using Wingate Anaerobic Tests (WAnTs). Prior to any testing, all participants attended a familiarization session to ensure they were familiar with the testing equipment and procedures.

Measurements began with lower body WAnT. Prior to the test, venous blood was taken at rest as well as at five and 30 minutes after completion. One day later, participants completed the upper limb WAnT, and blood samples were collected as previously described. For 48 hours prior to testing, participants were asked to refrain from exhaustive exercise, to maintain their normal dietary habits, and to come to the laboratory in euhydrated state.

### 2.4. Measurement of Anaerobic Fitness Components: Lower Body and Upper Body Wingate Tests

The lower body WAnT was conducted on a cycle ergometer (Monark 894E, Peak Bike, Sweden). For each participant, the saddle height was adjusted so the knee remained slightly flexed after the completion of the downward stroke (with final knee angle approximately 170–175°). Toe clips were used to ensure that the participants' feet were held firmly in place and in contact with the pedals. Before any experimental testing, each individual completed a standardised warm-up on the cycle ergometer (five min at 60 rpm, 1 W/kg). Each participant was required to pedal with maximum effort for a period of 30 s against a fixed resistive load of 75 g/kg of total body mass as recommended by Bar-Or [22].

The upper body WAnT was conducted on a hand cycle ergometer (Monark 891E). Participants sat in a chair fixed to the ground and were advised to keep their feet flat on the ground and remain seated throughout the WAnT. The seat height and backrest were adjusted so that, with the crank position on the opposite side to the body and the hand grasping the handles, the elbow joint was almost in full extension (140–155°), and the shoulders were in line with the centre of the ergometer's shaft. A standard resistive load equivalent to 50 g/kg of total body mass was applied for each participant [23]. Before the test, the participants completed a warm-up that involved five min of arm cranking using a power output of 1 W/kg and a crank rate of 60 rev/min.

For both lower and upper body WAnT, each participant was instructed to cycle as fast as possible and was given a three-second countdown before the set resistance was applied. Verbal encouragement was given to all participants to maintain their highest possible cadence throughout both WAnTs. Both cycle ergometers were connected to a PC to allow data capture via the MCE 5.1 software. The following WAnT variables were measured: peak power (W) and relative peak power (W/kg) were calculated as the highest single point of power output (recorded at 0.2 s intervals); mean power (W) and relative mean power (W/kg) were the average power output during the 30 s test; fatigue index (%) was the percentage of power loss determined for the time interval from the moment of obtaining the peak power by the end of the test

(1) $$\begin{array}{c} FI=\left(\;1-\frac{\int_{PPt}^{tf}P\left(t\right)\cdot dt}{PP\cdot \left(tf-PPt\right)}\;\right)\cdot 100\%, \end{array}$$

where FI is fatigue index, *t* is time, *P* is power, PP is peak power, PPt is time to peak power, and tf is time to finish the test.

### 2.5. Genetic Methods

Two ml of blood from renal vain was taken three times in each test: before, up to 5, and 30 min after the Wingate Tests. Each point of blood collection is marked in Figure 1.

For eliminated erythrocytes, Red Blood Cell Lysis (RBCL) buffer was used (five-part buffer to one-part blood), and obtained leukocytes were lysed using one ml Fenozol (A&A Biotechnology, Gdynia, Poland). For RNA isolation, the Chomczynski and Sacchi [24] protocol was applied. The quality and quantity of pure RNA were determined spectrophotometrically (Eppendorf BioPhotometer Plus, Germany). cDNA synthesis from 2 *μ*g RNA was performed using the TranscriptMe Kit containing oligo dT and random hexamers (Blirt, Gdańsk, Poland) according to the manufacturer's instruction. Real-time PCR (LightCycler 480 II, Roche, Poland) was performed two times in triplicate for each sample using a LightCycler polymerase (Roche, Poland). The temperature-time profile of the reaction was consistent with the manufacturer's instructions. For each reaction, a melt curve analysis was performed. The TATA box protein* (TBP)* and* S18* were used as housekeeping genes. Target genes were selected from five housekeeping genes experimentally:* ACTB*,* GAPDH*,* TUBB*,* TBP*, and* S18*. No changes in sample expression before and after exercise were observed for* TBP*,* TUUB*, and* S18*.

For analysis, the following primer sequences were applied:

 
*TBP* reverse: TCTGTCGGCTCCGCTCTGAGAT
 
*TBP* forward: ACTCCCGTTGTCCCAAGGCTTC
 
*S18* reverse: TTCCAATTACAGGGCCTCGAA
 
*S18 *forward: CGCAAATTACCCACTCCCG
 
*HSPA1A* reverse: TTCGGAGAGTTCTGGGATTGTA
 
*HSPA1A* forward: TGGACTGTTCTTCACTCTTGGC
 
*HSPB1* reverse: GAGGAAACTTGGGTGGGGTCCA
 
*HSPB1* forward: AAGGATGGCGTGGTGGAGATCA
 
*IL6* reverse: GACATCAAGGCGCATGTGAAC
 
*IL6* forward: TCCACGGCCTTGCTCTTGTTT
 
*IL10* reverse: AATTCGGTACATCCTCGACGG
 
*IL10* forward: GAATCCAGATTGGAAGCATCC
  HSF1 reverse: CAGGAGCTTGGAGTCCATGCA
  HSF1 forward: GAGCAGCTCCTTGAGAACATC
  NF-kB reverse: GATCCCATCCTCACAGTGTTT
  NF-kB forward: TGGACTACCTGGTGCCTCTA

### 2.6. Statistical Analysis

Descriptive statistics include mean ± SD for all measured variables. The normality of distribution was checked with Shapiro-Wilk's test. Gene expression data were collected and relative gene expression was analysed using Excel 2010. In order to calculate the level of gene expression, the method of Schmittgen and Livak [25] was used, and data were then transformed from logarithm 2 (2^∧^) to a linear value. To assess statistical significance of gene expression changes before and after exercise (WAnT), a repeated measures analysis of variance (ANOVA) was used. To determine the differences in gene expression after lower and upper body WAnT, a paired *t*-test was calculated. To calculate differences between values before and after the training period, two-way ANOVA of repeated measures (two groups × three measures) was applied. Post hoc analyses were implemented when appropriate with Tukey's post hoc test. The data was presented on the figures as 2^∧^fold changes (2^∧^FC). In addition, the effect size of the researched relations was estimated (Cohen's *d* values). All calculations and graphics were performed using GraphPad Prism 6.0 (ftx.pl/program/graphpad-prism). Differences were considered statistically significant at a level of *p* ≤ 0.05.

## 3. Results

### 3.1. Effect of Eight Weeks of HIIT to Relative Mean and Peak Power

Eight weeks of HIIT caused a significant increase in maximal power (722 to 751 W after training, 4% increase) and relative peak power (10.1 to 11 W/kg after training, 3.8% increase) in lower body WAnTs. In the upper body, significant changes were observed for anaerobic possibilities in mean power (from 444 to 464 W, 4.5% increase), relative mean power (from 6.5 to 6.8 W/kg, 4.6% increase), and the fatigue index (from 27.7 to 24.2%, 12.6% decrease). Before and after training, all parameters were significantly higher in lower body WAnTs, excluding the fatigue index (Table 2).

### 3.2. Gene Expression Changes in Lower and Upper Body Exercise before the Training Period

**Figure 2:**
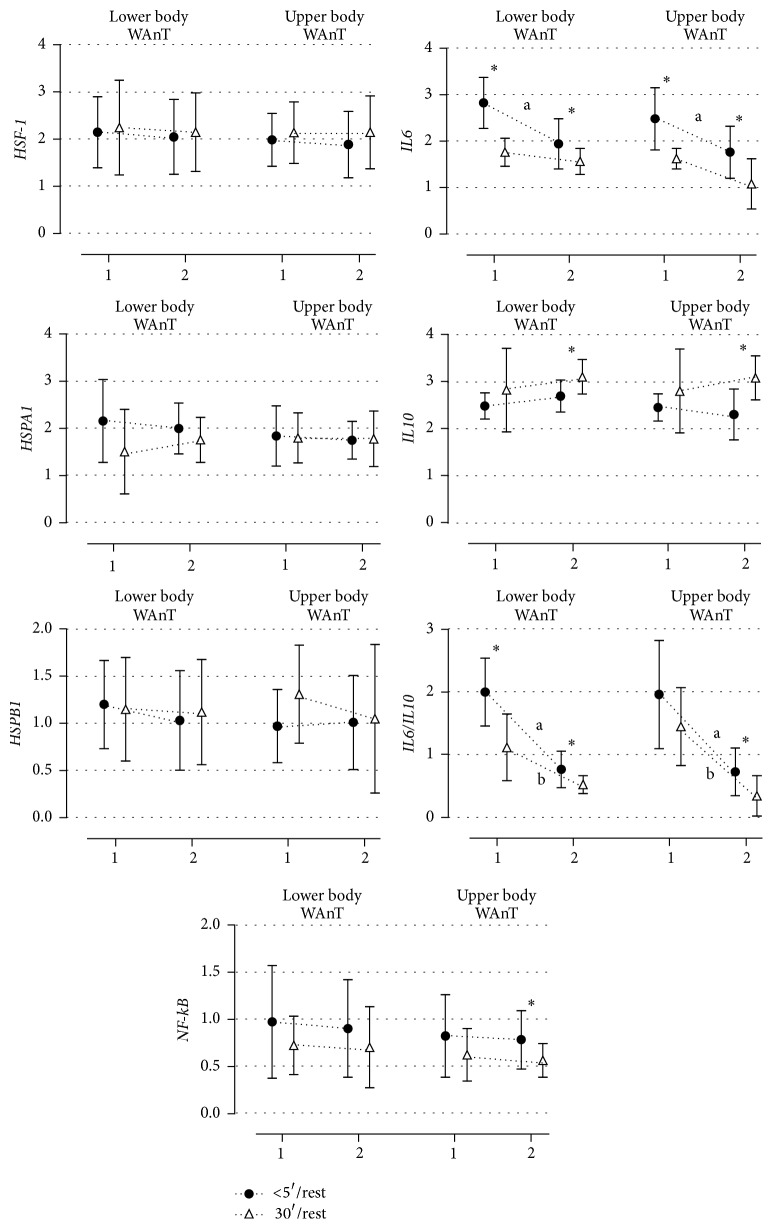
Changes in relative expression up to five min and 30 min after lower and upper body Wingate Test before (1) and after (2) training period. ^*∗*^Significant expression differences between up to 5 and 30 min after exercises. (a) Significant differences between 1 and 2 experiments performed by the lower body. (b) Significant differences between 1 and 2 experiments performed by the upper body.

Changes in gene expression at both five and 30 min after laboratory tests are presented in Figure 2. There were no significant differences in gene expression between lower and upper body Wingate at either time points. Mean* HSF-1* and* IL-10* mRNA was slightly increased 30 min after lower and upper body exercises, respectively, while* HSPA1A*,* NF-kB*,* IL-6* mRNA, and the* IL6/IL10* ratio was decreased. Additionally, a time effect was seen in the* IL6* mRNA and IL6/IL10 ratio. Significantly lower expression of this gene was observed 30 min after exercise performed by the lower body (changes from 2^∧^2.82 5 min after to 2^∧^1.76-fold 30 min after, *p* = 0.0001) and the upper body (changes from 2^∧^2.48 5 min after to 2^∧^1.62-fold 30 min after exercise, *p* = 0.0019).* IL6/IL10* ratio decreased from 2^∧^1.99 5 min after to 2^∧^1.11 30 min after exercise *p* = 0.0019 performed by lower body exercise and from 2^∧^1.95 5 min after to 2^∧^1.44 30 min *p* = 0.05 after upper body (*p* = 0.05).

Eight weeks of HIIT showed no differences between lower and upper body WAnTs. However, gene expression was changed at two time points, five and 30 min after exercise.* HSF-1* and* HSPB1* mRNA was slightly higher 30 min after exercises, and* IL-10* mRNA was significantly higher (*p* < 0.05) while* HSPA1A* and the* IL6/IL10* mRNA ratio was slightly lower in the same time.* IL-6 *mRNA (*p* < 0.05) was significantly lower for this time point, similar to observation made in Experiment 1. In detail changes between five and 30 min after WAnTs for* Il6* mRNA were from 2^∧^1.94 to 2^∧^1.56-fold, *p* = 0.03 after lower body, and from 1.76 to 2^∧^1.08, *p* = 0.02 after upper body exercises. The increase for* IL-10* mRNA from 2^∧^2.69 to 2^∧^3.10 *p* = 0.01 after lower body and from 2^∧^2.30 to 2^∧^ 3.08 fold *p* = 0.03 after upper body WAnT was observed in the same time.

Eight weeks of training caused no changes in response to the Wingate Test, with the exception of the IL6/IL10 ratio. Irrespective of significance, the direction of changes observed in* HSF-1*,* HSPA1A,* and* NF-kB* mRNA was similar.* HSPA1A*,* HSF-1, *and* NF-kB* mRNA was lower immediately after exercise following eight weeks of intensity training. Similarly, no significant changes in* NF-kB*,* IL-6,* and* I-L10* mRNA were observed in response to the Wingate Test. Changes of* IL6*,* IL10,* and* NF-kB* mRNA were associated with decreased* NF-kB* and* IL6*-mRNA expression and increased* IL10* mRNA (5 and 30 min after WAnTs, resp.). Therefore, the decrease in the* IL-6/IL-10* ratio was significant after lower (−2^∧^0.57, *p* = 0.0019) and upper body exercises (−2^∧^0.5, *p* = 0.03).

## 4. Discussion

When examining the expression of genes associated with the cellular stress response, this is the first study comparing the differences between lower and upper body adaptive effects caused by eight weeks of HIIT. It is known that changes in gene mRNA are dependent on many factors: specificity of muscular activity [26]; the different character of physical effort involving slow twitch or fast twitch fibers [27]; various types of training [28]; and differences between sedentary and active people [29]. It is also possible that such changes are associated with specific types of training that accompany various sporting disciplines [30].

Changes in anaerobic capacity after eight weeks of HIIT in lower and upper body exercises were a reflection of technical skills used in the applied training period. Significant differences in peak power and relative peak power (lower body WAnT), as well as mean, relative mean power, and fatigue index (upper body WAnT), are mainly associated with different training loads implemented on particular instruments. Earlier studies demonstrated that, in gymnasts, peak power in the lower body is more crucial in acrobatic elements or jumps. Instruments involving the lower body, lasting 30–45 s, require mean power, relative mean power, and muscle resistance to fatigue. HIIT programs can induce greater fatigue as well as higher ratings of perceived exertion compared with the classical moderate-intensity continuous training [31, 32]. In our study, after six weeks of HIIT, the index fatigue significantly decreased after upper body exercise.

There are only two studies in which anaerobic capabilities were investigated in gymnasts. Jemni et al. [33] showed that, in elite French gymnasts, relative peak power was 13.4 ± 1.3 W/kg and mean power was 9.6 ± 1.0 W/kg. These parameters were higher in the lower body than in the upper body, and they were higher than the results observed in our athletes. Similarly, higher relative peak results (11.7 ± 1.2 W/kg) were also observed in elite Greek gymnasts [34]. In contrast, anaerobic capabilities of the upper body in our athletes appeared similar to those described in the above-mentioned French gymnasts (according to Jemni et al. [33]: relative peak power: 9.2 ± 1.1 W/kg; relative mean power: 6.6 ± 0.6 W/kg) as well as to judo athletes [19] and wrestling [35]. No data in the literature from HIIT in gymnasts are available.

After WAnTs were performed, all tested genes were upregulated at both 5 and 30 min after applied exercises. Our hypothesis stated that gene expression changes after upper body exercise were similar to those obtained for the lower body because of systemic responses to exercises. In our research the changes in genes expression were evaluated in leukocytes, the cells with high antioxidant capacity [36]. According to Żychowska et al. [16] and Maltseva et al. [15] changes in genes expression in these cells show a systemic response to exercise. Results confirmed that, before and after the applied training period, gene expression differences between lower and upper body exercises were not significant. The results indicated similar stresses for leukocytes after lower and upper body exercises, despite the fact that lower body exercises involved higher muscle mass. According to Szołtysek et al. [11], the cellular stress response is dependent on activation of* HSF-1* and* NF-kB* pathways. Regardless of the kind of stressors, the cellular stress response is the result of this activation. In this study, there were no differences in* HSF-1* and* NF-kB* expression between lower and upper body exercises. Therefore, it is possible that there was a similar total stress load after lower and upper body exercises (including oxidative stress, changes in temperature, and muscle damage, which can influence the expression of tested genes).

Eight weeks of HIIT caused no significant changes in the expression of* HSF-1*,* HSPA1A,* and* HSPB1 *mRNA in response to lower and upper body exercises. The adaptive changes to exercise were evident in lower* HSPA1A *expression. It is possible that eight weeks of training was insufficient for high level athletes in which adaptive changes to exercises are not very dynamic [15, 30]; therefore, eight weeks of training may be too short for further adaptive changes in these genes. Cytokines, including interleukin-6 (IL-6) and interleukin-10 (IL-10), are key agents of the immune system and are involved in the systemic response to local inflammation [4, 37]. In our study, significant differences were observed for* IL-6* and* Il-10* in response to lower and upper body WAnTs after eight weeks of HIIT. Decreased expression of* NF-kB* and* IL6* mRNA was seen while increased* IL10* mRNA was measured. This was marked by a significant decrease in the IL6/IL10 ratio after exercises performed by the lower and upper body. It is known that adaptive changes to exercises involve decreases in* IL6* and increases in* IL-10* mRNA [17]. Decreased* NF-kB* activation could be caused by increased* IL10* mRNA [38, 39]. Additionally, HIE performed before and after the training period caused decreased* IL6 *and increased* IL10* mRNA in a time dependent manner (either five or 30 min after exercises). However, after the eight-week training period, these differences were higher at both time points after exercise and caused a significant decrease in the* IL6/IL10* ratio. Our results are similar to those reported by Nieman et al. [40] and Kaspar et al. [4]. The authors observed time dependent expression of* IL10* and* IL6* after WAnT (2.7-fold increase immediately after for* IL10* and 0.8-fold decrease one hour after for* IL6* mRNA). Two hours after WAnT,* IL-6* mRNA returned to the rest value [40]. Kaspar et al. [4] observed a significant decrease in the IL6/IL10 ratio from baseline to 30 minutes after exercise (−20%) in seven untrained (active) people who performed HIE. Our data confirm this observation, as 30 min following exercise, the changes in the IL6/IL10 mRNA ratio were the same.

Zwetsloot et al. [1] suggested that HIIT exercise induces a small inflammatory response in young, recreationally active men; however, two weeks of HIIT does not alter this response. In our study, eight weeks of HIIT caused significant changes in gene expression of* NF-kB*,* IL6, *and* IL10*. This difference may be associated with several factors such as applied training load and duration of training (higher in the gymnasts of our study). Thus, our study provides several novel findings. HIE causes upregulation of genes associated with the cellular stress response, and eight weeks of HIIT does not alter this response in* HSF-1*,* HSPA1A,* and* HSPB1* mRNA. The significantly lower expression of* IL6* and higher expression of* IL10* after eight weeks of HIIT indicated this kind of training is effective to acquisition and maintenance adaptive changes to intensity exercises. Finally, all indicated adaptive changes can be obtained by lower and upper body exercise. This is important, not only for athletes, but also for all people in which lower body exercise is limited. Furthermore, in higher level athletes the adaptive changes to exercises on molecular level are not very dynamic and did not affect any further adaptation changes in genes encoding HSP.

## Figures and Tables

**Table 1:** One specific week of training applied for eight weeks.

Day	Gymnastics training	Exercise	Duration-effectiveness time (s)	Avg Hr
Monday	04:30–07:00 p.m.	HIAP	230	155
		TS	500–850	125
Tuesday	7:20–9:20 a.m. 03.00–05.00 p.m.	HIAP	230–235	155
		TS	500–850	125
Wednesday	7:20–9:20 a.m. 03:00–05:00 p.m.	HIAP	230–240	155
		TS	500–850	125
Thursday	03:00–05:00 p.m.	BR	600–850	
Friday	7:20–9:20 a.m. 03:00–05:00 p.m.	HIAP	230	155
		TS	500–850	125
Saturday	09:00–12:00 a.m.	HIAP	230–250	155
		TS	500	125
Sunday	free day			

High intensity anaerobic performance (HIAP); technical skills (TS); biological renewal (BR).

**Table 2:** Lower and upper body Wingate Anaerobic Test (WAnT) characteristics of athletes before and after eight weeks of HIIT.

	I		II		% change	Cohen's *d*
	Mean ± SD	(95% CI)	Mean ± SD	(95% CI)		
*Lower body WAnT*						
Maximal power (W)	722 ± 113^∧^	657–788	751 ± 124^*∗*∧^	679–822	4.0	0.24
Mean power (W)	562 ± 73^∧^	520–605	577 ± 80^∧^	531–624	2,7	0.19
Relative peak power (W/kg)	10.6 ± 0.8^∧^	10.1–11.1	11.0 ± 1.0^*∗*^	10.4–11.5	3.8	0.44
Relative mean power (W/kg)	8.2 ± 0.5^∧^	8.0–8.5	8.4 ± 0.5^∧^	8.2–8.7	2.4	0.40
Fatigue index (%)	25.6 ± 6.5	21.8–29.4	26.2 ± 5.9	22.8–29.6	2.3	0.09
*Upper body WAnT*						
Maximal power (W)	575 ± 123	504–647	586 ± 120	517–656	1.9	0.09
Mean Power (W)	444 ± 84	395–493	464 ± 86^†^	414–513	4.5	0.23
Relative peak power (W/kg)	8.4 ± 1.3	7.7–9.2	8.6 ± 1.3	7.8–9.2	2.4	0.15
Relative mean power (W/kg)	6.5 ± 0.8	6.0–7.0	6.8 ± 0.9^†^	6.2–7.3	4.6	0.35
Fatigue index (%)	27.7 ± 6.0	24.2–31.1	24.2 ± 5.7^*∗*^	20.9–27.5	−12.6	0.59

^*∗*^ *p* < 0.05, ^†^*p* < 0.005, significant difference in lower and upper body WanTs before (I) and after (II) eight weeks of HIIT; ^∧^*p* < 0.005, significant difference between the lower and upper body WanT in a particular measurement (I or II).
